# Percutaneous pelvic ring fracture reduction using an external fixator: a technical trick and case series

**DOI:** 10.1007/s00264-025-06509-0

**Published:** 2025-04-02

**Authors:** Guillaume David, Nicholas J. Tucker, Clement Marc, Vincent Steiger, Louis Rony, Cyril Mauffrey

**Affiliations:** 1 Centre Hospitalier Universitaire d’Angers, Angers, France; 2 Denver Health Medical Center, Denver, USA

**Keywords:** Percutaneous pelvic ring fracture reduction, External fixator, Minimally invasive technique, Pelvic trauma, Reduction technique

## Abstract

**Purpose:**

Pelvic ring and acetabular fractures pose significant morbidity and mortality risks due to substantial haemorrhage and internal organ injury. Many percutaneous reduction techniques involve manipulating the injured side while stabilizing the uninjured side, often requiring specific or costly equipment. This article presents a technique for creating a pelvic reduction frame using a standard external fixator.

**Method:**

We included surgical pelvic ring fractures between 2018 and 2022. Pelvic reduction was achieved using an external fixator (Hoffmann III, Stryker Corporation, Kalamazoo, Michigan, USA). Reduction quality was assessed according to the technique described by Lefaivre et al., based on the following criteria: mean asymmetry (mm), mean deformity index (mm), and mean maximum horizontal or vertical displacement (mm).

**Results:**

15 patients (10 men, 5 women, mean age 35 years) underwent surgical treatment for pelvic fractures using an external fixator and percutaneous fixation. Mean operative time was 130 min (range, 80–276). Postoperative imaging showed a mean maximum displacement of 5.4 mm and a mean asymmetry of 3.7 mm, with excellent or good reductions in 11 cases.

**Conclusion:**

This system uses widely available equipment and enables the benefits of percutaneous techniques, but surgical expertise remains the key to success.

## Introduction

Pelvic ring and acetabular fractures are accompanied by high morbidity and mortality due to frequently associated haemorrhage and injury to internal organs [1, 2]. In addition, the treatment of these fractures can be a real challenge for the orthopaedic trauma surgeon due to the complexity of fracture patterns and high-risk structures surrounding these regions [3]. Enabled by simultaneous advancements in techniques and intraoperative imaging, percutaneous pelvic surgery techniques emerged in the early 1990s. Initially limited to the sacroiliac joint, authors have since described new techniques for anterior and posterior stabilization of the pelvic ring with excellent clinical outcomes [4, 5]. The benefits of minimally invasive pelvic procedures include limiting soft tissue dissection, minimal intraoperative blood loss, shorter procedure times, and faster patient mobilization [6]. Many authors have described percutaneous reduction techniques using methods based on manipulating the injured side while stabilizing the uninjured side [7–10]. A major challenge in these cases, however, is maintaining reduction to allow for stable percutaneous fixation. To address this, many authors have published on a variety of techniques that often require specific and sometimes expensive equipment [11]. In this study, we present a step-by-step technique and case series of results using a standard external fixator to create an accessible, effective pelvic reduction system for pelvic fracture management.

## Materials and methods

### Population

This prospective study included 15 patients who underwent surgical treatment for pelvic ring fractures between 2018 and 2022. Patients were selected based on the need for operative reduction and fixation. Demographic data, including age, sex, and BMI, were recorded. Fractures were classified according to the Young and Burgess system into Lateral Compression (LC), Antero-Posterior Compression (APC), and Vertical Shear injury patterns.

### Preoperative planning

A thorough preoperative evaluation of plain radiographs and cross-sectional imaging is critical for achieving satisfactory reduction of pelvic ring fractures. This analysis identifies both primary fractures and associated injuries. Surgical planning includes assessing displacement on standard radiographs (anteroposterior, inlet, and outlet views), as well as on computed tomography (CT) scans and three-dimensional reconstructions. Preoperative assessment also aims to identify specific morphologies or injury characteristics that must be considered during surgical planning, such as sacral dysmorphism or crescent fractures.

### Positioning, system alignment, reduction, and fixation

The patient is positioned on a radiolucent flattop (Flat Jackson) table with the injured leg placed in the surgical field for manipulation and to facilitate the placement of distalfemoral skeletal traction, if necessary, to apply continuous axial traction using simple weights. The pelvic reduction system utilizes a large-caliber external fixator designed for femoral or pelvic applications (Hoffmann III, Stryker Corporation, Kalamazoo, Michigan, USA), along with the manufacturer’s largest pins, which are 6 mm in diameter and 250 mm in length.

**Fig. 1:**
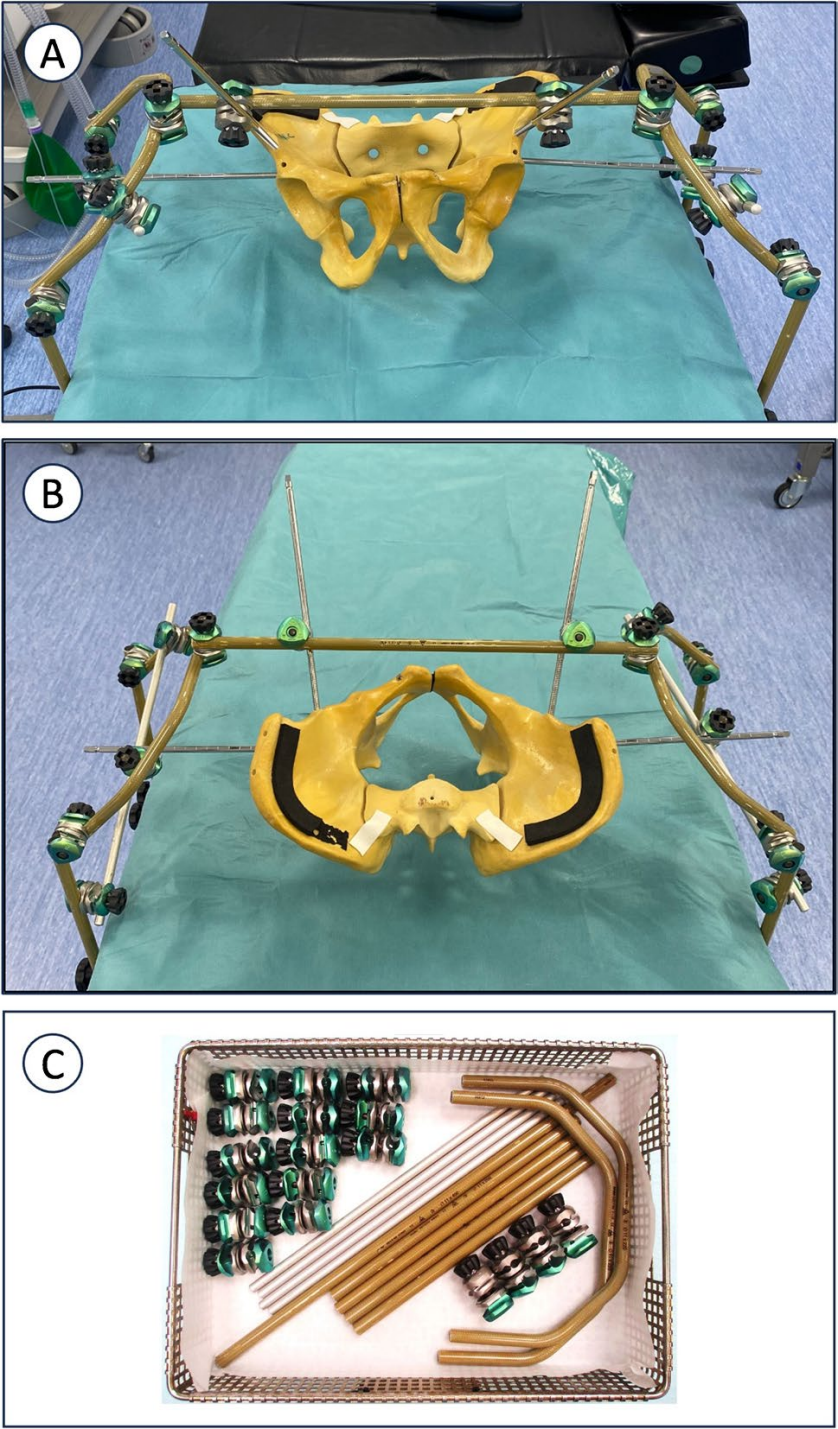
Clinical photograph of frame construction using external fixator bars and clamps

The first stage involves placing pins in both innominate bones, on the injured and uninjured sides. Each hemipelvis is secured with a double orthogonal fixation: a supra-acetabular pin in the frontal plane and a pin in the LC2 (lateral compression type 2) corridor in the sagittal plane (Figs. 1a – 1b). This technique provides enhanced control of cranial/caudal reduction, as described in the literature, and improves rotational stability [12]. The pins are set using the following technique with the necessary fluoroscopic views. [13] The supra-acetabular pin in the frontal plane is placed above the sourcil of the acetabulum using a standard anteroposterior (AP) view until it reaches the internal table of the innominate bone, ensuring the joint is not violated. For the LC2 pin, a teardrop or 'Teepee' view (outlet obturator) can be used to visualize the corridor (although not necessary to obtain as can interfere with instrumentation). Given the pin length and the challenge of aligning the corridor axis under fluoroscopy, two orthogonal views are employed. The iliac view allows visualization of the entry point at the anterior inferior iliac spine (AIIS) and guides the cranio-caudal trajectory with the surgeon aiming just cranial to the greater sciatic notch. The inlet obturator view is used to ensure pin placement is not intra- or extrapelvic.

**Fig. 2:**
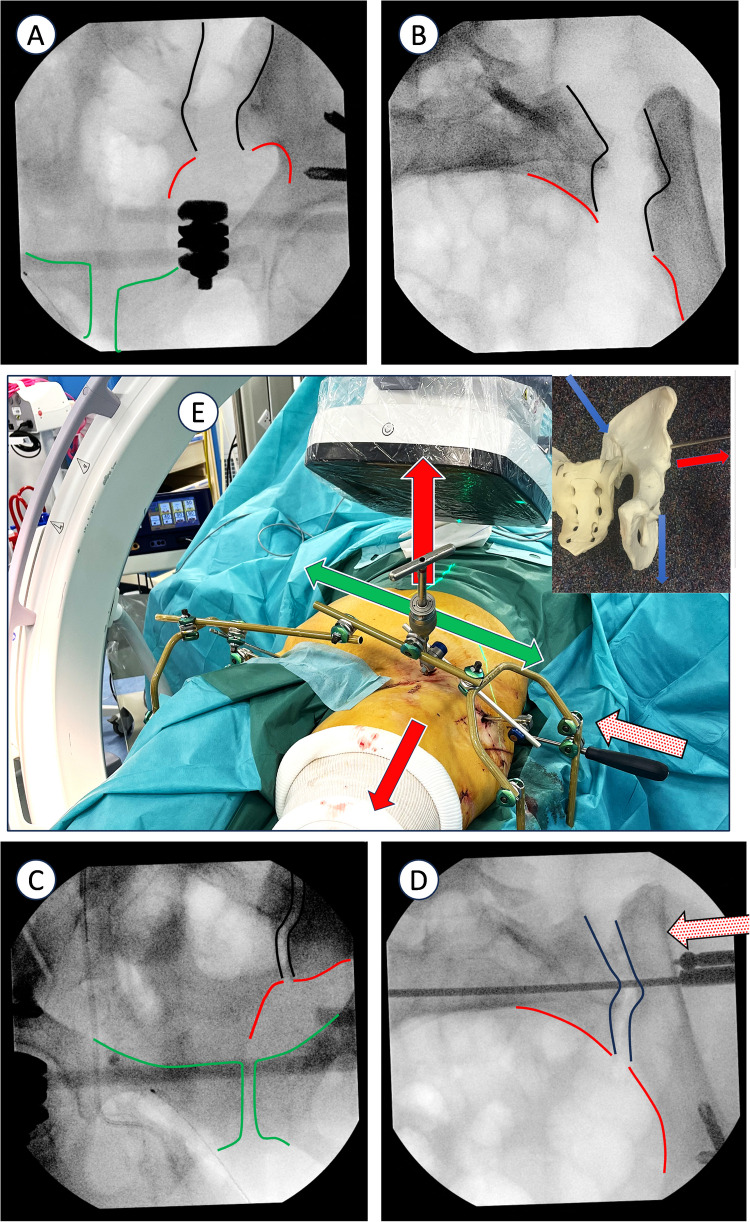
Case example of wide SI joint corrected with the technique of frame construction to close down SI joint diastasis

The fixation frame is then assembled around the patient. The most comprehensive version of the setup is shown in Fig. 1, though lighter versions may be used depending on the injuries being treated. The right and left sections of the system are connected by a crossbar to enhance frame stability. The uninjured side of the pelvis is 'locked' into the adjacent frame section, allowing for more effective manipulation of the injured side. To achieve this, key radiological landmarks must be identified. In the inlet view, these include the symphysis (anterior and posterior aspects), the anterior portion of the sacroiliac joint (inner table of the sciatic buttress on one side and sacral ala on the other), and the anterior wall of the sacral vertebrae. In the outlet view, landmarks include the symphysis (upper pubic ramus), the greater sciatic notches and the curve they form with the sacrum, the cephalad portion of the sacroiliac joint, and the iliac crests. In addition to the standard inlet and outlet views, specific views such as the inlet obturator view ('down-the-sacroiliac-joint view') provide clear visualization of the sacroiliac joint and the outer table of the iliac tuberosity, enabling more precise control over the placement and length of iliosacral (IS) or transsacral (TS) screws (Fig. 2) [4].

Axial traction is often necessary due to cranial displacement, and the placement of a T-handle on the LC2 pin aids in managing anteroposterior translation and rotation of the injured pelvis (the typical deformity involves external rotation of the hemipelvis with posterior translation relative to the intact sacrum). Varus/valgus alignment of the injured pelvis is controlled via the frontal supraacetabular pin. When satisfactory reduction of one parameter is achieved, the corresponding pin is secured to the frame, allowing additional reduction manoeuvres to be performed until a satisfactory reduction is achieved. For example, a ball spike pusher may be useful to close sacroiliac joint diastasis (Fig. 2). Additionally, in cases where posterior translation persists, one could suspend a Schanz pin toward the ceiling and secure it to the frame post, allowing the patient’s weight/gravity to correct the posterior hemi-translation gradually.

**Fig. 3:**
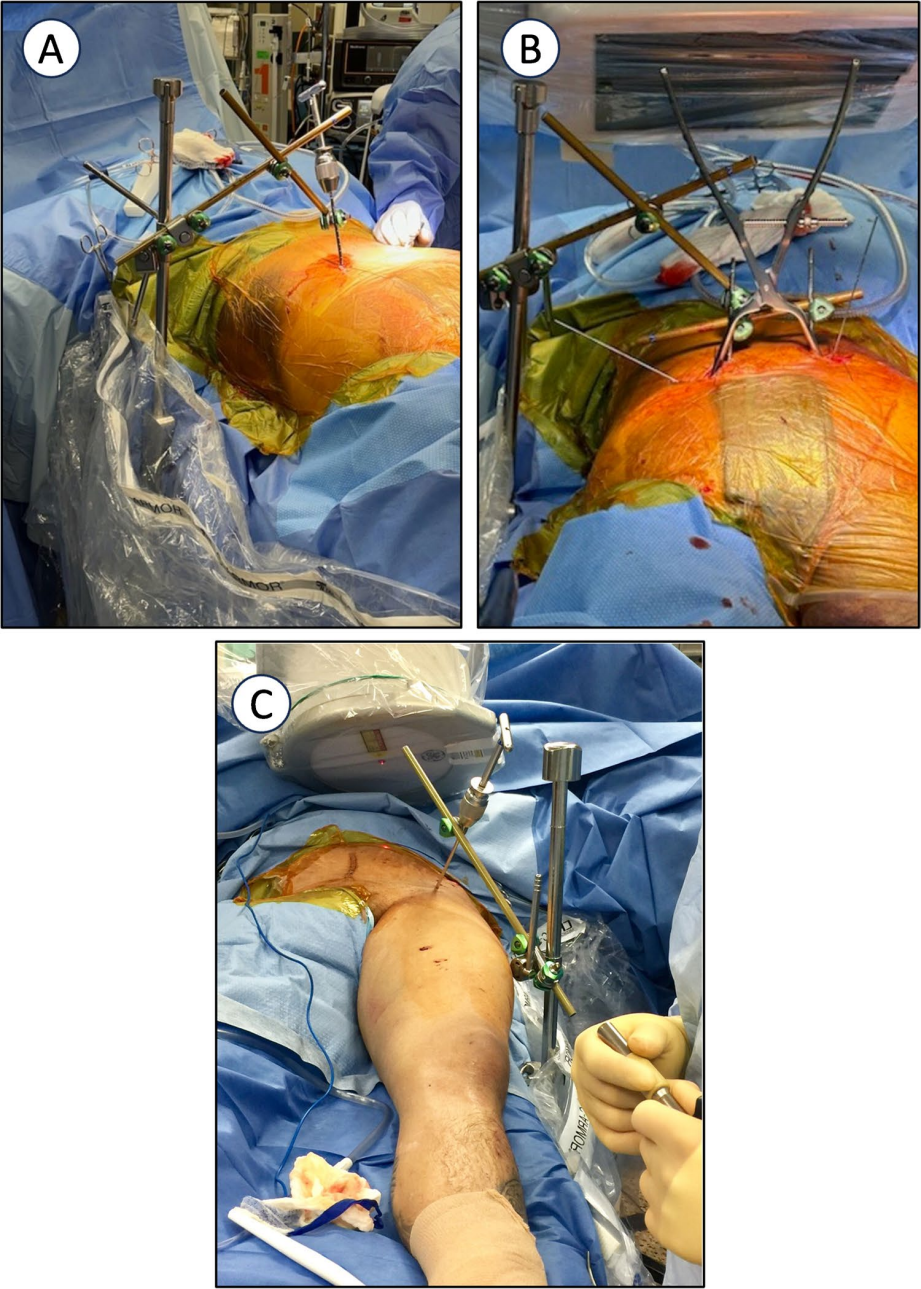
Clinical picture of the use of a Thompson retractor post to build the frame on a prone patient with significant posterior pelvic ring injury. The clamp is placed on the posterior ilium to compress the posterior ring and held with a posterior pelvic ring external fixator

This technique can also be applied with the patient in the prone position, using a minimally invasive approach to the SI joint. A Thompson retractor can also be used to anchor the system to the operating table. (Fig. 3).

**Fig. 4:**
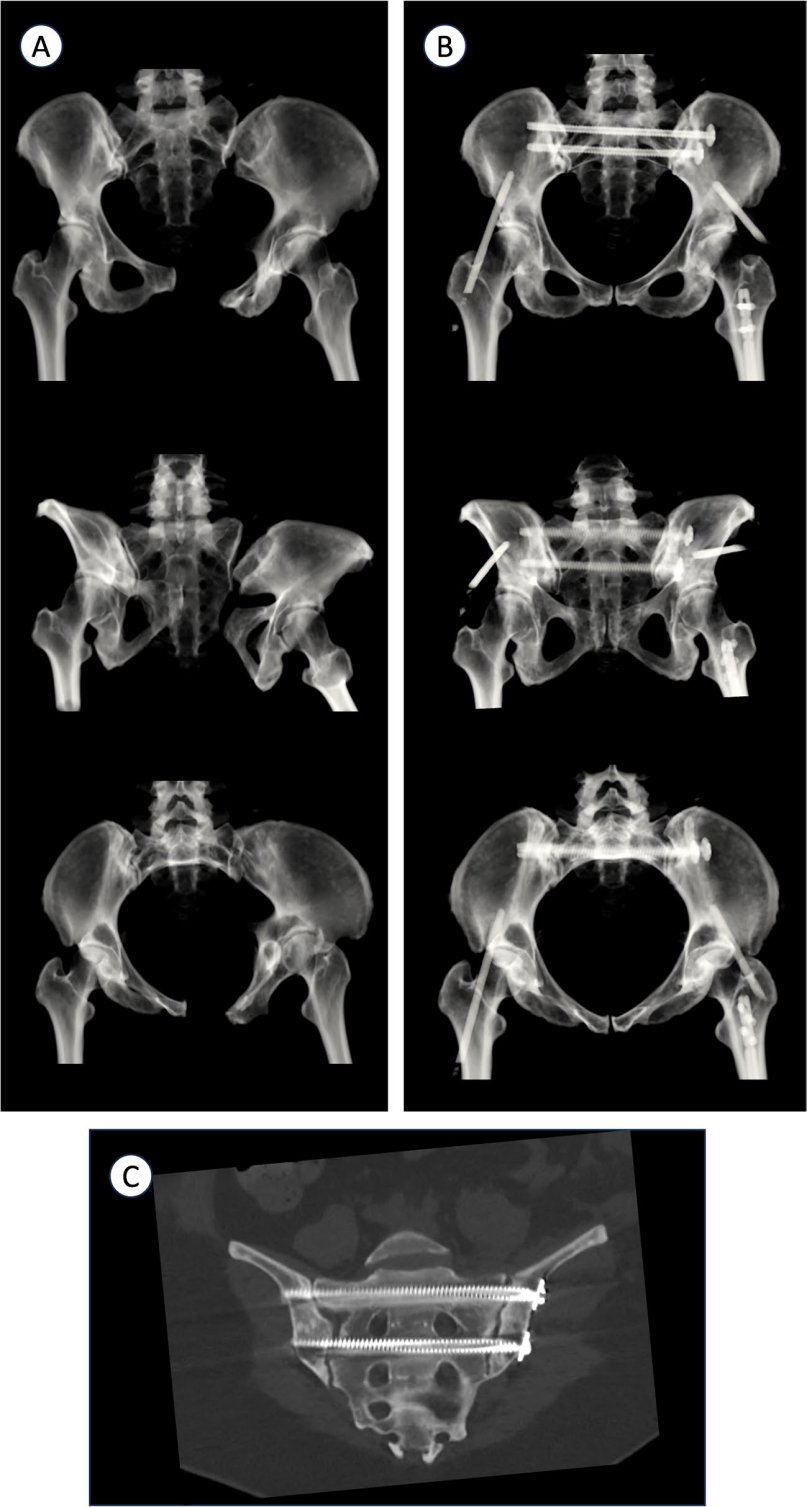
Postoperative CT scan demonstrating reduction and screws trajectories

Once the reduction is considered satisfactory, appropriate percutaneous fixation can be performed utilizing techniques well-described in the literature.(Fig. 4).

### Postoperative protocol

A postoperative CT scan is routinely performed to evaluate fracture reduction and implant placement. Touchdown weight-bearing is typically recommended for patients on the affected side unless prevented by other injuries. First postoperative follow-up is scheduled at six weeks for a clinical and radiological assessment to check for any fracture displacement or hardware issues. Additional follow-up is arranged if there are any existing concerns about wound healing. Follow-up radiographs are then taken at three months, six months, and one year to monitor fracture healing and any potential development of post-traumatic arthritis.

## Results

A total of 15 pelvic fractures treated operatively using an external fixator connected to the operative table between 2018 and 2022 were included in the study. The cohort consisted of ten men and five women with a mean age of 35 years (range, 23–54 years). Average BMI was 30 (range, 21.2–40.6). Fracture types were categorized as follows: Lateral-Compression (LC) type 3 fractures: five cases; Antero-Posterior Compression (APC) type 3 fractures: four cases; LC type 2 fractures: two cases; and Vertical Shear injuries: four cases. Mean operative time was 130 min (range, 80–276 min). No postoperative infections were recorded. Postoperative radiographs (AP, Inlet, Outlet views) and CT scans were reviewed to assess residual displacement. Measurements followed techniques previously described in similar studies [14]. Using this approach, an excellent reduction was achieved in four patients, a good reduction in seven patients, and a fair reduction in four patients. The mean maximum postoperative absolute displacement was 5.4 mm, and the mean asymmetry on the AP view was 3.7 mm. Maximum displacement and correction gains are detailed in the Table 1. Overall, a satisfactory correction was achieved in a majority of cases.

**Table 1:** Maximum displacement on pre- and post-operative anteroposterior pelvic radiographs

Measurement	Pre-operatively	Post-operatively	Correction obtained
Mean asymmetry (X–Y) in mm (range)	17.8 ± 11,7 (4,9—43,7)	3,74 ± 4,99 (0,15—17,58)	14,08 ± 12,29 (2,73—42,66)
Mean deformity index (X–Y/X + Y) (range)	0,063 ± 0,041 (0,018—0,133)	0,015 ± 0,022 (0,0007—0,08)	0,048 ± 0,042 (0,01—0,128)
Mean maximum horizontal or vertical displacement in mm (range)	17,63 ± 9,46 (2,03—37,86)	5,38 ± 8,8 (0,34—32,49)	12,26 ± 10,48 (1,13—36,74)

## Discussion

While open reduction and internal fixation has historically been considered the gold standard for treating displaced pelvis and acetabulum fractures, it is often associated with significant complications and potential for blood loss [15–18]. Percutaneous techniques, being quicker and less invasive, were initially considered appropriate mainly for non-displaced fractures. However, with the advancement of understanding and execution of new percutaneous reduction techniques, percutaneous screw fixation methods are increasingly gaining popularity [19].

Pelvic and acetabular fractures, with increasing prevalence, pose significant technical and financial challenges due to their complexity and extended hospital stays [20, 21]. Modern minimally invasive techniques address these issues by reducing complication risks and shortening recovery times, thereby lowering overall costs. In today’s financially constrained healthcare environment, adopting such cost-effective solutions is crucial for balancing effective care with budgetary constraints, helping to manage resources while improving patient outcomes. Using equipment available in all Level 1 trauma centres, this technique does not require additional or specialized tools specifically for pelvic surgery but rather relies on equipment commonly used in routine trauma care. Additionally, the simplicity of its use allows a trained orthopaedic pelvic surgeon to adapt effectively to the specific characteristics of each fracture type being treated.

While this system offers significant benefits, it cannot compensate for a surgeon's technical shortcomings. A deep understanding of pelvic anatomy and fracture mechanics is crucial for effective use of this technique to achieve a desired reduction and surgical fixation. Surgeons must possess both detailed knowledge and technical skill to manage these complex fractures effectively. These techniques also require a perfect understanding of pelvic radioanatomy [22]. The use of intraoperative CT can be a valuable asset in reducing technical failures and iatrogenic complications [23].

In conclusion, this cost-effective and readily available pelvic reduction system utilizing an external fixator provides a useful tool/technique to achieve and maintain pelvic ring reductions for use with percutaneous fixation methods. As its use necessitates an in-depth knowledge of pelvic surgery, it is again important to emphasize that this approach and technique cannot compensate for insufficient surgical expertise.

## Data Availability

No datasets were generated or analysed during the current study.
